# Risk of gastrointestinal bleeding by specific SSRIs and SNRIs: A systematic review and meta‐analysis

**DOI:** 10.1002/bcp.70432

**Published:** 2025-12-29

**Authors:** Ainhoa Gomez‐Lumbreras, Abdelrahman G. Tawfik, Guilherme Del Fiol, Kensaku Kawamoto, Thomas Reese, Katy Trinkley, Aubrey Jones, James Mitchell, Daniel C. Malone

**Affiliations:** ^1^ Department of Pharmacotherapy, College of Pharmacy University of Utah Salt Lake City Utah USA; ^2^ Department of Biomedical Informatics, School of Medicine University of Utah Salt Lake City Utah USA; ^3^ Department of Biomedical Informatics Vanderbilt University Nashville Tennessee USA; ^4^ School of Medicine University of Colorado Anschutz Medical Campus Aurora Colorado USA

**Keywords:** antidepressive agents, gastrointestinal haemorrhage, selective serotonin reuptake inhibitors, serotonin and noradrenaline reuptake inhibitors

## Abstract

**Aim:**

The purpose of this study is to estimate the risk of gastrointestinal bleeding (GIB) by selective serotonin reuptake inhibitors (SSRIs) and serotonin–norepinephrine reuptake inhibitors (SNRIs) individual agents.

**Methods:**

A systematic review was conducted for each unique antidepressant (i.e. SSRI: citalopram, escitalopram, fluoxetine, fluvoxamine, paroxetine, and sertraline; and SNRIs: desvenlafaxine, venlafaxine, and duloxetine) combined with search terms for GIB in PubMed and EMBASE from inception to October 2025. Articles including results on specific antidepressants and GIB risk were included.

**Results:**

From a total of 1218 identified publications, 20 studies were included and analysed using a random‐effect meta‐analysis. Twelve studies (60%) used a case–control design, three (15%) a cohort study design, one (5%) a case cross‐over, one (5%) used both case–control and cross‐over designs and three (15%) were randomized control trials (RCTs). Studies sample sizes ranged from 666 235 from a Medicaid population to 1280 from 43 hospitals participating in a RCT. Fluoxetine had the most studies providing evidence (19 studies) and fluvoxamine and duloxetine had the least (five studies). Each antidepressant showed an increased risk of GIB. Venlafaxine had the highest estimated risk (OR 1.50, 95% CI 1.32–1.70), followed by citalopram (OR 1.38, 95% CI 1.17–1.62) and fluoxetine (OR 1.38, 95% CI 1.26–1.51). Paroxetine had the lowest GIB risk (OR 1.31, 95% CI 1.07–1.62).

**Conclusion:**

GIB is an uncommon adverse event, but this analysis demonstrates that the risk of GIB is elevated for commonly used SSRI/SNRI products, highlighting the relevance for those patients with an increased risk of GIB.

## 1 INTRODUCTION

In the United States, more than 10% of adults have used antidepressants, and more than 20% among older than 60 years old, similar to European countries.^1^, ^2^, ^3^ Because of improved safety and tolerability, selective serotonin reuptake inhibitors (SSRIs) and serotonin‐norepinephrine reuptake inhibitors (SNRIs) are preferred agents.^1^, ^4^, ^5^, ^6^, ^7^ However, SSRIs and SNRIs are not exempt of harms and have been associated with other serious adverse events.^7^, ^8^ One of the most serious harms is haemorrhage, especially when occurring in the gastrointestinal tract (GI), intracranial (IC), postpartum, or post‐surgical.^9^, ^10^, ^11^, ^12^, ^13^ Bleeding events at these sites can be life‐threatening. Previous studies on the class effects of SSRIs and SNRIs have reported an increased risk of haemorrhage from 40% to 51%.^10^, ^12^, ^13^

SSRIs and SNRIs inhibit the reuptake of serotonin via inhibition of the serotonin transporter (SERT) receptor of the monoamine transporter subfamily.^14^ The underlying mechanism for the risk of haemorrhage is inhibiting serotonin storage in platelets.^15^ Pharmacokinetic studies have established different degrees of serotonin inhibition and of dissociation constants for the serotonin transporter for each antidepressant.^16^, ^17^ Several studies have attempted to associate the degree of serotonin reuptake inhibition and bleeding risk.^18^, ^19^, ^20^, ^21^ Another underlying mechanism, in the case of gastrointestinal bleeding (GIB), is that SSRIs increase acid secretion, directly leading to ulcerogenic effects.^13^

Safety information for antidepressant agents has been reported in randomized clinical trials (RCTs) but has several limitations. GIB is not frequently reported as an adverse event because it is an uncommon outcome or because reported gastrointestinal ‘events’ are not well‐described, making it challenging to evaluate GIB events. Previously, observational studies have analysed the data at the therapeutic class level (e.g. SSRI or SNRI).^22^, ^23^ Also, some studies have used vague definitions for bleeding events with no specification regarding the site.^24^ Meta‐analyses that have been conducted examined the type of bleeding (e.g. GI, IC, post‐surgery, etc.), but these reports analyse the data at the therapeutic class level.^12^, ^19^, ^25^, ^26^ While pharmacodynamic studies suggest GIB varies by individual agent, the comparative impact of the individual SSRIs and SNRI on GIB has not been studied. Thus, the purpose of this systematic review and meta‐analysis was to examine the risk of GIB for each specific SSRI and SNRI.

## 2 METHODS

We conducted a systematic review of the literature and meta‐analysis on GIB risk associated with individual SSRI/SNRI following the Preferred Reporting Items for Systematic reviews and Meta‐Analyses (PRISMA) guideline.^27^ See Data S1.

### 2.1 Search strategy

We conducted a literature search using PubMed and EMBASE databases. For PubMed, the MeSH term “*Gastrointestinal Haemorrhage*” and free text (gastrointestinal bleed*) were combined with each of the following SSRI antidepressants (i.e., citalopram, escitalopram, fluoxetine, fluvoxamine, paroxetine and sertraline) and SNRIs (desvenlafaxine, venlafaxine and duloxetine). EMBASE database was queried following a similar approach. Timeline for both databases was from inception to October 2025. Furthermore, included studies were evaluated to identify additional cited articles via PubMed and Web of Science. See search strategy in Data S2. Meta‐analysis identified using the search strategies were reviewed to identify other potential articles.

### 2.2 Inclusion and exclusion criteria

Eligibility criteria of the studies were evaluated following the PICO (Population, Intervention, Comparison, Outcome) framework.^28^ Inclusion criteria for articles required information specific to specific antidepressant agents and GIB. If antidepressants were grouped by mechanism of action (e.g. SSRI, SNRI, monoamine oxidase inhibitors, tricyclic antidepressants, etc.), we did not include these studies, with the exception of those studies that grouped the antidepressants by their degree of affinity for the serotonin transporter. If several time exposures were assessed (e.g. < 7 days, <30 days, >60 days … etc.), the shortest duration since starting the medication was used. Comparisons of interest were monotherapy antidepressant evaluated relative to a placebo/no treatment. Composite endpoints or other definitions (e.g. major bleeding) that included GIB, but not the isolated outcome of GIB, were excluded. Articles that reported bleeding but did not specify the site were excluded.

Systematic reviews and meta‐analyses were prioritized followed by RCTs and then observational evidence to gather information on the quantification of the GIB risk for the different antidepressant agents.^29^ Case reports, case series, and non‐quantitative review articles were excluded. Measures of risk [odds ratio (OR), hazard ratio (HR), relative risk ratio (RR) or the number of events] were required for a study to be included.

### 2.3 Data extraction and quality assessment

AGL and AGT independently reviewed all the titles and abstracts provided by the search strategy. After title and abstract screening, full text articles were independently reviewed, and if disagreement for inclusion, consensus was reached through discussion among AGL, AGT and DCM. From the selected studies, we examined the citations of those studies and used the “*Cited by*” filter in PubMed and Web of Science. AGL, AGT and DCM reviewed the data of interest to verify accuracy and extract the correct estimates. AGL and AGT performed a cross‐reference search with all the references cited by the selected articles.

The quality of the included studies was assessed using either the Newcastle Ottawa Scale (NOS) for observational studies or the Cochrane Risk of Bias 2 Tool (RoB 2) for clinical trials.^30^, ^31^ *For the NOS*, the assessed domains included patient selection, comparability of studies and assessment of outcomes. Studies scoring six or higher on the NOS were considered to be of high quality.

### 2.4 Analysis

Standardized risk estimates presented as RRs, ORs or HRs with corresponding 95% confidence intervals (CI) were used.^32^ To minimize confounding, covariate‐adjusted estimates were used when available; unadjusted estimates were used when adjusted estimates were not reported. We used a random‐effects meta‐analysis to pool risk estimates, providing pooled OR with 95% CIs for each antidepressant for the risk of GIB.^33^ To assess publication bias, Egger's test was conducted for each antidepressant. Statistical analyses were performed using the R software (R4.5.0).

Key protein targets and ligands in this article are hyperlinked to corresponding entries in http://www.guidetopharmacology.org, and are permanently archived in the Concise Guide to PHARMACOLOGY 2021/2022.^34^

## 3 RESULTS

**FIGURE 1:**
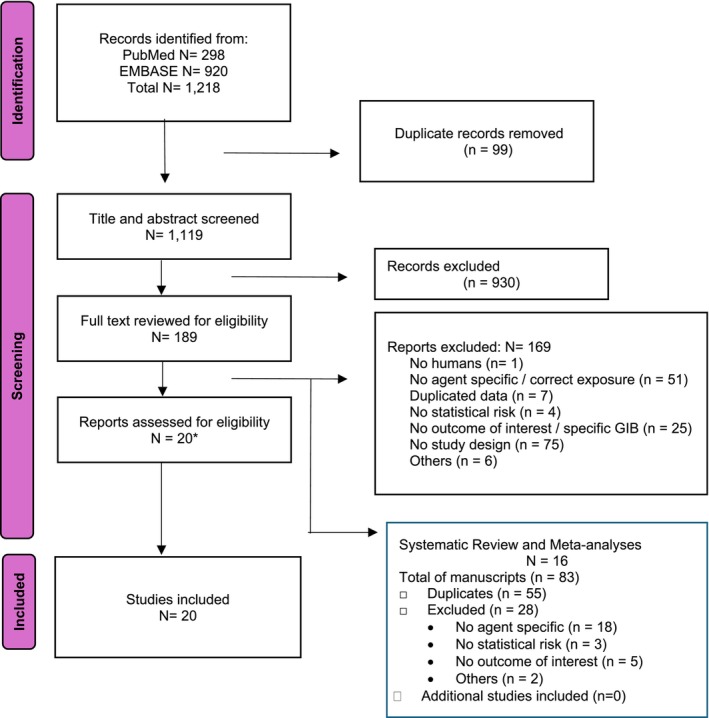
PRISMA flowchart of the study selection. *The included studies were subsequently used to perform a citation analysis using the Web of Science database and PubMed. The selection process is shown in the Data S2.

From a total of 1218 studies, after removal of 99 duplicates, a total of 1119 titles were identified. After title and abstract screening, a total of 189 full texts were reviewed, with a total of 20 manuscripts selected to be included in the meta‐analysis (Figure 1).^21^, ^35^, ^36^, ^37^, ^38^, ^39^, ^40^, ^41^, ^42^, ^43^, ^44^, ^45^, ^46^, ^47^, ^48^, ^49^, ^50^, ^51^, ^52^, ^53^ The included studies were 17 (85.0%) observational studies and three (15.0%) RCT on fluoxetine. Among the observational studies, 12 (60.0%) were case control studies,^21^, ^35^, ^36^, ^37^, ^40^, ^41^, ^42^, ^43^, ^44^, ^46^, ^47^, ^52^ three (15.0%) had a cohort study design,^38^, ^48^, ^53^ one (5.0%) used a case‐crossover design,^45^ and one (5.0%) used both the case–control and the case cross‐over analysis.^39^ A description of the selected studies for the evidence on GIB risk estimated for each unique antidepressant is shown in Table 1. The observational studies included data from 1993 (De Abajo et al., 1999) to 2020 (Forgerini et al.).^40^, ^52^ The study settings were mainly from Europe (*N* = 12),^21^, ^35^, ^36^, ^37^, ^39^, ^40^, ^41^, ^43^, ^48^, ^49^, ^50^, ^53^ three in North America,^42^, ^44^, ^46^ one in South America,^52^ and four in Asia,^38^, ^45^, ^47^, ^51^ with the AFFINITY trial being conducted in Australia and New Zeland.^51^ The observational studies from Asia analysed data from the Taiwan National Health Insurance Research Database (NHIRD).^38^, ^45^, ^47^

**TABLE 1:** Characteristics of included studies.

Study	Design	Data source/setting	Study period/length	Risk metric	Exposure definition	GIB definition	Sample size	Population characteristics		
								Age	Male (%)	Race (%)
AFFINITY trial^51^	RCT, parallel‐group, double‐blind, placebo control	43 hospitals: in Australia (*n* = 29), New Zealand (four), and Vietnam (10)	11 Jan 2013 to 30 June 2019/ 6 years	OR (95% CI)	Fluoxetine 20 mg capsules or matching placebo capsules	Adverse events: UGIB	1280	Mean exposed 63.5 (SD 12.5); Mean control 64.6 (SD 12.2)	Exposed 64%; Control 62%	Exposed: Asian 55%; White 42%; Others 3%; Control: Asian 58%, White 40%, Other 2%
Barbui 2009^36^	Case–control	ARNO database (Italian local health units). Italy	1 January 2003 to 31 December 2005/3 years	OR_adj_ (95% CI)	Any prescriptions within 180 days before the index hospital admission compared with no use of antidepressants	ICD‐9 codes. First admission for bleeding abnormalities	2998	All ages (0 = 18, 19–44, 45–64, ≥65)	64.4	NA
Carvajal 2011^37^	Case–control	Spain (four hospitals) and Italy (one hospital)	January 2004 to July 2006 (Spain)/2 years and 7 months and October 2005 to November 2007 (Italy)/2 years and 2 months	OR_adj_ (95% CI)	Use of antidepressants until the index (bled) date or when discontinued within 7 days before the index date	Admitted patients with a primary diagnosis of acute UGIB from a duodenal or gastric ulcer, acute lesions of the gastric mucosa, erosive duodenitis, or mixed lesions, all of them diagnosed by endoscopy	1939	≥18; Mean 62.6 (SD 17.0)	71.3	NA
Chang 2022^38^	Cohort	Taiwan National Health Insurance Research Database (NHIRD)	1 June 2012 to 31 December 2017/5 years and 6 months	OR_adj_ (95% CI)	DOAC prescriptions for more than 28 days, and antidepressants prescribed longer than 28 days in each person‐quarter	ICD‐9 and ICD‐10 codes	98 863	≥30 to ≤105; Mean 74.9 (SD 10.3)	56.2	NA
Coupland 2018^48^	Cohort	QResearch primary‐care research database. United Kingdom	1 January 2000 until 31 July 2011/11.5 years	HR_adj_ (95% CI)	Prescriptions for antidepressants. Continually exposed: no >90 days between the end of one prescription and the start of the next	Read codes and ICD codes. Exclusion of patients who had had an upper GI bleed by the baseline date	238 963	Range:20–64 Mean 39.5 (SD 11.1)	38.9	White/non‐ recorded (95.2) and non‐White (4.8)
Dall 2009^39^	Case–control case‐cross over	Danish Databases: ‐Funen County Patient Administrative System (FPAS) ‐Odense University Pharmaco‐epidemiological Database (OPED) ‐Danish Central Person Register (CPR)	August 1, 1995 to July 31, 2006 / 11 years	OR_adj_ (95% CI)	Days equivalent to the number of units dispensed plus 20%	ICD‐8 and ICD‐10 codes	40 154	All (<55, ≥55 and <75, ≥75); Mean 72.1 (SD 14.1)	51.2	NA
De Abajo 1999^40^	Case–control	General Practice Research Database (GPRD) United Kingdom	April 1993 to September 1997/ 4 years and 5 months	OR_adj_ (95% CI)	‘*Current users*’ prescription for antidepressants lasted until index date or ended within 30 days of the index date	UGIB or ulcer perforation (OXMIS code for UGIB) + manual review of computerized profiles	11 651	40–79	NA	NA
De Abajo 2008^41^	Case–control	The Health Improvement Network (THIN) database. United Kingdom	January 2000 to December 2005/6 years	OR_adj_ (95% CI)	‘C*urrent users*’ prescribed antidepressants until index date or were discontinued within 30 days of the index date	READ codes and manually extracted by computer search	11 321	40–85	56.9	NA
EFFECTS trial^50^	RCT, parallel‐group, double‐blind, placebo control	35 centres in Sweden	20 October 2014 and 28 June 2019	OR (95% CI)	Fluoxetine 20 mg or matching placebo	Adverse events: UGIB	1500	Mean exposed 70.6 (SD 11.3); mean control 71.0 (SD 10.5)	Exposed and control 62%;	Exposed: White 99%; control: 98%
FOCUS trial^49^	Pragmatic RCT, parallel‐group, double‐blind, placebo control	103 hospitals in the UK	10 September 2012 and 31 March 2017	OR (95% CI)	Fluoxetine 20 mg or placebo	Adverse events: UGIB	3127	Mean exposeed: 71.2 (SD 12.4); mean control 71.5 (SD 12.1)	Exp 62%; control 61%	Exposed and control: Asian 2%; Black 2%; White 96%;
Forgerini 2023^52^	Case–control	Clinical Hospital of the Ribeirão Preto Medical School of the University of São Paulo, Brazil	July 2016 to March 2020	OR (95% CI)	Pharmaceutical anamnesis was performed for all participants used during the preceding 2 months and electronic medical record	Gastroduodenal lesions and erosions, peptic ulcers (gastric and duodenal) or mixed lesions associated with clinical symptoms (e.g. hematemesis, dark vomiting or in ‘coffee‐grounds,’ melena, and/or haematochezia) and confirmed by endoscopy within 48 h of hospital admission or laparoscopy	906	Cases 60.2 (16.3); controls 59.8 (SD 15.8)	Cases 72.5; controls 72.5	Cases White 71.5, mixed 16.5, Black 11.5 and Asian 0.5; controls White 76.8, Mixed 14.4, Black 8.2 and Asian 0.6
Kurdyak 2005^42^	Case–control	Three databases in Ontario, Canada: the Ontario Drug Benefit Program, the Canadian Institute for Health Information Discharge Abstract Database, and the Ontario Registered Persons Database	January 1994 to December 2002/9 years	RR_adj_ (95% CI)	Antidepressants within 90 days before admission for UGIB	ICD‐9 codes	16 734	≥66 Mean 80.3 (SD 6.8)	51	NA
Li 2024^47^	Case–control	Taiwan National Health Insurance program	1 January 2000 and 31 December 2013	OR_adj_ (95% CI)	At least two prescriptions of SSRI	UGIB ICD 9 codes. Exclusion of patients with previous UGIB	2512	79.0 (SD 6.0)	340 (54)	NA
Magavern 2023^53^	Cohort	Genes and Health (Barts Health NHS Trust). UK	16 September 2014	OR (95% CI)	One or more prescription for an antidepressant, used chronically	ICD10 codes	22 753	Non‐AD 39 (±14); AD 46 (±14)	Non‐AD 38; AD 32	NA
Opatrny 2008^43^	Case–control	General Practice Research Database (GPRD) in the United Kingdom	2000 to 2005/6 years	Rate ratio_adj_ (95% CI)	Prescription 90 days prior to the index date	First diagnosis of upper GIB identified using a READ/OMXIS medical code. All cases with a first diagnosis of upper GI haemorrhage	44 199	Mean 69.3 (SD 17.6)	43.9	NA
Schelleman 2011^44^	Case–control	Medicaid data of California, Florida, New York, Ohio, and Pennsylvania	1999 to 2005 /7 years	OR_adj_ (95% CI)	Antidepressant claim within 29 days prior to or on the index‐date	ICD‐9 codes	666 235	≥18	36.5	59.5% Caucasian 14.5% African American; 26.0% Other/ Unknown
Verdel 2011^35^	Case–control	PHARMO RLS Netherlands	January 1998 to December 2007/10 years	OR_adj_ (95% CI)	Prescription within 90 days of the index date	ICD‐9 codes	24 844	≥18	52.3	NA
Vidal 2008^21^	Case–control	Spain (ten) and in Italy (eight)	September 1998 to December 2001 (Spain)/3 years and 3 months and November 1999 to December 2001 (Italy)/2 years and a month	OR_adj_ (95% CI)	Use in the 7 days before the index day	Endoscopic confirmed GIB	10 006	≥18	NA	NA
Wang 2014^45^	Case‐crossover study	National Health Insurance Research Database (NHIRD). Taiwan	1998–2009/11 years	OR_adj_ (95% CI)	Short term (1–14 days before the index date) to patients who were exposed only during the 14‐day control period (15–28 days before the index date)	ICD‐9 codes	5377	≥20; Mean 57.6 (SD 19.9)	NA	NA
Wessinger 2006^46^	Case–control	Northwestern Memorial Hospital, Rush University Medical Center, Chicago, Illinois. Mayo Clinic Hospital, Phoenix, Arizona. US.	1 January 2003 to 1 April 2004/1 year and 3 months	OR (95% CI)	Outpatient medication reported at admission	ICD‐9 codes	1579	≥18; Cases mean:65.8 (SD 0.8)	49.6	60% Caucasian; 5.4% Hispanic; 27.9% African‐American; 6.6% Other

[Correction added on 12 January 2026, after first online publication: The reference numbers have been corrected in this version.]

Abbreviations: UGIB, upper gastrointestinal bleeding; HR, hazards ratio; ICD, International Classification of Diseases; ICPC, International Classification of Primary Care; OR, odds ratio; RR, relative risk/risk ratio; SD, standard deviation; NA, not available; RCT, randomized controlled trial.

For the observational studies, the NOS quality rating was of six or more stars (median 7.2, minimum‐maximum 6–9), with Coupland et al. having a perfect quality score of 9.^48^ Most studies missed the definition of controls as they did not specify that the outcome of interest was not present or had not been present; in the case of the cohort studies, they did not mention the outcome not being present at start.^38^, ^40^, ^41^, ^42^, ^43^, ^45^, ^47^, ^53^ (See complete NOS quality assessment in Tables S3A and S3B). The RoB score was low for the AFFINITY and EFFECTS trials, but scored some concerns for the FOCUS trial (see Data S3C).^49^, ^50^, ^51^

With respect to the population of interest and sample size, Schelleman et al. evaluated 666 235 patients receiving warfarin and Coupland et al. a cohort of 238 963 patients with depression.^44^, ^48^ Combined, these two studies represent the majority of evidence. Schelleman et al. used Medicaid claims data from 1999 to 2005 across five states: California, Florida, New York, Ohio and Pennsylvania.^44^ Coupland et al. analysed the QResearch, a primary‐care research database from United Kingdom over 12 million patients.^48^

Forgerini et al., Schelleman et al., Coupland et al. and Wessinger et al. were the only studies including information on race, with 71%, 59.5%, 95.2% and 60% Caucasian/White in each study, respectively.^44^, ^46^, ^48^, ^52^ The average age for those studies with no age limit criteria was greater than 65 years of age.^36^, ^39^ Coupland et al. and Opatrny et al. reported as male 38.9% and 43.9% of their population, respectively.^43^, ^48^ The remainder of the studies population were majority male, with Carvajal et al. reporting 71.3% of male and Forgerini 72.5%.^37^, ^52^ De Abajo et al., 1999 did not report information on the sex or age of the population, and Vidal et al. did not provide data on the sex of the studied population.^21^, ^40^ Only three RCTs were included that studied fluoxetine 20 mg *vs*. placebo over 6 months.^49^, ^50^, ^51^ The populations were similar, with almost 1500 participants each RCT with 60% males and White (over 95%).

### 3.1 Exposure definition

The description for antidepressant exposure varied across the studies, as it is shown in Table 1. When assessing the time of exposure, Verdel et al., Schelleman et al., Opatrny et al., and Barbui et al. described exposure as a prescription prior to the index date, with different time windows for exposures (e.g. 0–29 days, 30–59 days and 60–119 days).^35^, ^36^, ^43^, ^44^ De Abajo et al. considered current exposure when the prescription ended within 30 days of the index date for both the reported results.^40^, ^41^ Coupland et al. and Carvajal et al. conducted sub‐analyses varying the time/duration of exposure (i.e. previous month, 30–59 days and 60 to 119 days).^37^, ^48^ Wessinger et al. defined exposed if the antidepressant was reported at admission of the bleeding event.^46^ Forgerini et al. retrieve information from participants and also from electronic medical records in the 2 months preceding the GIB event.^52^

Four studies grouped antidepressant agents by the degree of affinity for the serotonin transporter in high, medium or low affinity.^21^, ^35^, ^37^, ^52^ Vidal et al., Carvajal et al. and Forgerini et al. grouped the antidepressants according to the results provided in the study of Tatsumi et al.,^21^, ^37^, ^52^, ^54^ and Verdel et al. provided the risks based on the degree of affinity according to the Psychoactive Drug Screening Program database.^35^, ^55^ However, there were discrepancies regarding the classification of medium‐ and high‐affinity drugs: Verdel et al. classified fluvoxamine, citalopram and duloxetine as high affinity, whereas Carvajal et al. and Forgerini et al. classified them as medium affinity.^35^, ^37^, ^52^ Vidal et al. estimated risk based on the level of serotonin reuptake inhibition too, but analysed each specific antidepressant agent GIB risk.^21^

The dose of antidepressant was only indicated in RCTs studying fluoxetine (i.e. fluoxetine 20 mg capsules) and accounted by Li et al. using defined daily doses.^47^, ^49^, ^50^, ^51^ The remainder of studies included no information on dose.

### 3.2 Outcome definition

The definition of GIB event was not homogeneous as shown in Table 1. Forgerini et al., Vidal et al. and Carvajal et al. used endoscopy diagnosis.^21^, ^37^, ^52^ Both studies by de Abajo et al. identified computer profiles using OXMIS and READ codes for upper GIB, as did Opatrny et al.^40^, ^41^, ^43^ However, both de Abajo et al. manually conducted a chart review of the selected cases.^40^, ^41^ The remaining observational studies (*N* = 11, 55%) used codes from the International Classification of Diseases (ICD) different editions, 8th or 9th or 10th.^35^, ^36^, ^38^, ^39^, ^42^, ^44^, ^45^, ^46^, ^47^, ^48^, ^53^ Dall et al. reviewed over 12 000 discharge summaries.^39^ Barbui et al. specified that the GIB had to be present on the first admission for bleeding, and Coupland et al. excluded 1600 patients with a prior upper GIB.^36^, ^48^

### 3.3 Meta‐analysis: risk of GIB

**FIGURE 2:**
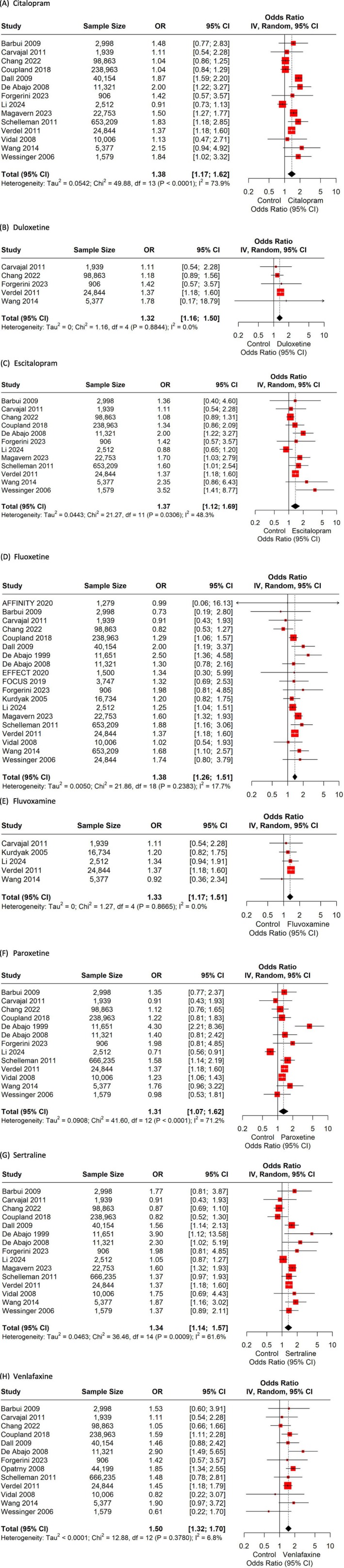
Risk of gastrointestinal bleeding by selective serotonin reuptake inhibitors and selective norepinephrine reuptake inhibitors.

Displayed in Figure 2 are the results from the meta‐analysis for each agent included in the study. All antidepressants significantly increase in the risk of GIB as compared to no antidepressant treatment or placebo.

Citalopram was evaluated in 14 studies,^21^, ^35^, ^36^, ^37^, ^38^, ^39^, ^41^, ^44^, ^45^, ^46^, ^47^, ^48^, ^52^, ^53^ with de Abajo et al. (2008) and Wang et al. showing a risk of 2 (OR_adj_ 2.0, 95% CI 1.2–3.4 and OR_adj_ 2.15, 95% CI 0.94–4.92, respectively) (Figure 2A).^41^, ^45^ Its enantiomer, escitalopram, had a similar point estimate of GIB risk (Figure 2C).^56^ Three studies considered citalopram and escitalopram grouped: Carvajal et al., De Abajo et al. and Verdel et al.^35^, ^37^, ^41^ In our meta‐analysis, their results were similar, with OR 1.38 (95% CI 1.17–1.62, I^2^ 73.9%) for citalopram and OR 1.37 (95% CI 1.12–1.69, I^2^ 48.3%) for escitalopram.

Duloxetine and fluvoxamine had fewer studies, and only Verdel et al. found a significant risk (OR 1.37_adj_, 95% CI 1.18–1.60).^35^ Both agents showed similar risk of GIB in our meta‐analysis (OR 1.32, 95% CI 1.16–1.50 for duloxetine and OR 1.33, 95% CI 1.17–1.51 for fluvoxamine) (Figure 2B and E, respectively).

Fluoxetine was included in all studies except for the Opatrny et al. (Figure 2D).^43^ The GIB risk ranged from 2.50 (De Abajo et al. 1999) to 0.73 (Barbui et al.) across the 19 studies.^36^, ^40^ The risk of GIB for fluoxetine was 1.38 (95% CI: 1.26–1.51; Figure 2D). Paroxetine GIB risk estimate was 1.31 (95%CI 1.07–1.62), with a high heterogeneity (*I* ^2^ = 71.2%). Sertraline risk was 1.34 (95% CI 1.14–1.57) and not that high heterogeneity, *I* ^2^ = 61.6%. (Figure 2F and G, respectively). Both antidepressants included the same studies except sertraline, which also included Dall et al. and Magavern et al.^39^, ^53^

Opatrny et al., using the General Practice Research Database, evaluated venlafaxine independently from the other antidepressants that were grouped according the mechanism (i.e. SSRIs, TCAs).^43^ Our meta‐analysis showed an OR of 1.50 (95% CI 1.32–1.70) for venlafaxine (Figure 2H) with low heterogeneity (*I* ^2^ = 6.8%). No studies on desvenlafaxine were identified.

### 3.4 Risk of publication bias

The risk of publication bias was assessed with Egger's test and funnel plots (see Data S4). Egger's test showed no evidence of publication bias, and for some agents with few studies (duloxetine *N* = 5, fluvoxamine *N* = 5), the Egger's test could not be performed.

## 4 DISCUSSION

This systematic review and meta‐analyses included 20 studies and found that specific SSRIs/SNRIs are associated with an increased risk of GIB (ranging from 31% to a 50%). However, we found no significant differences between the products as all 95% CI intervals overlapped. Because of differences in the degree of serotonin inhibition across products, we expected differences in clinical outcomes as well.^15^, ^57^, ^58^

Previous systematic reviews and meta‐analyses have documented the risk of GIB for antidepressants at the therapeutic class level (i.e. SSRI, TCA or SNRI).^59^, ^60^, ^61^, ^62^ In an era of precision medicine, tailoring medical treatment to individual characteristics is needed to optimize effectiveness and reduce risk of harm.^63^ For patients with high risk of GIB, clinicians may believe that one treatment is safer than others, but this analysis suggests there is no difference with respect to GIB risk and that current guidelines referencing the antidepressant class are appropriate.^64^, ^65^, ^66^

The studies included in our analysis were mainly observational case–control designs, as this design is frequently used to assess risk of harm for rare outcomes, such as GIB. Pivotal RCTs evaluating antidepressants do not commonly report data on GIB and often classify adverse events based on the organ affected (gastrointestinal side effects) or nonspecific bleeding.^67^ Large RCTs have not been conducted to study risk of GIB.^68^, ^69^, ^70^ There is some evidence from RCT for duloxetine where Perahia et al. conducted an analysis with individual patient data from over 50 placebo‐controlled post‐marketing trials to assess the risk of bleeding.^71^ However, the study did not provide data on GIB. The United States Food and Drug Administration product labels for these products mention “*Abnormal bleeding*” and “*Bleeding reactions related to SNRIs and SSRIs use have ranged from ecchymoses, hematomas, epistaxis, and petechiae to life‐threatening hemorrhages*,” but no specific information on GIB is mentioned in product labelling.^72^, ^73^, ^74^ Post hoc analyses of the direct acting oral anticoagulant pivotal studies assessing the risk of bleeding when concomitantly exposed to antidepressants do not report results for unique antidepressant agents either.^75^, ^76^

Other research reported results for bleeding that was not site specific or agent specific.^24^, ^61^, ^77^, ^78^, ^79^ GIB is a rare outcome (78 per 100 000 person per year, ranging from 15 to 172 per 100 000 person years in an over 30 years systematic review of GIB incidence).^80^, ^81^ Various studies have included GIB, perforation and obstruction as peptic ulcer complications (PUC), or peptic ulcer disease or gastrointestinal toxicity.^60^, ^82^ Studies have validated that when assessing PUC, most of the events are bleeding, with around 10% being perforation and around 85% bleeding events.^83^, ^84^, ^85^ There are several meta‐analysis that have focused on the potential interaction between nonsteroidal anti‐inflammatory drugs (NSAIDs) and SSRI increasing the risk of GIB.^59^, ^77^, ^79^ The mechanism by which NSAIDs cause GIB is through inhibition of cyclooxygenase enzymes that affect platelet clot formation and decrease prostaglandins that protect the GI tract, usually resulting in higher rates of GIB events among SSRI/NSAIDs users.

Despite guidance on reporting of sociodemographic characteristics for observation studies,^86^ the age of the studied cohort was not reported in five (25%) studies,^21^, ^35^, ^40^, ^41^, ^44^ sex was absent in three (15%),^21^, ^40^, ^45^ and race was only reported in seven (35%).^44^, ^46^, ^48^, ^49^, ^50^, ^51^, ^52^ Concerns on the representation in clinical trials have already been highlighted because of lack of generalizability.^87^, ^88^, ^89^ Whether this could be fulfilled with observational data is debated.^90^ None of the selected studies included specific risk estimates for desvenlafaxine. Therefore, one could assume that the risk estimate would be the same as venlafaxine, as desvenlafaxine is the active metabolite of venlafaxine.^91^

### 4.1 Limitations

A limitation of this study is that 85% of the included studies were observational studies which may have misclassified the exposure and the outcome, as previously identified for some observational research.^92^ The definitions of exposure to antidepressants and of the outcome of interest (GIB) varied across the included studies. Another limitation is that information on the dose was not available and could not be incorporated into analysis. The exception is Li et al., who compared the high *vs*. low dose of the antidepressants, and three RCTs that focussed specifically on fluoxetine 20 mg per day.^47^, ^49^, ^50^, ^51^ We did not assume the risk estimate would be uniform across the studies and, therefore, used a random‐effect model in our meta‐analysis to account for studies heterogeneity. However, this may not remove underlying systematic differences that arise with observational studies. Selection bias could be present in the included studies; therefore, when available, we conducted our analyses using the multi‐variate adjusted results for the GIB risk estimates.

This study is limited to SSRI and SNRI products. We did not include other antidepressants that have some affinity to serotonin transporter, such as clomipramine, imipramine and amitriptyline, as these are not frequently used.^1^, ^54^, ^93^ These products were not frequently used, likely because of the perceived safety profile of SSRI and SNRI agents.^94^ We also did not include antidepressants with different mechanisms of action, such as bupropion or agomelatine, as these agents mechanism of action would not imply an increase on the risk of GIB.^95^

Although we conducted a wide and comprehensive search, it is possible that relevant studies were not identified. However, a total of 16 meta‐analysis on risk of GIB and antidepressants identified from the PubMed (N = 13) and EMBASE (N = 3) search strategies were reviewed.^10^, ^13^, ^19^, ^24^, ^59^, ^61^, ^62^, ^77^, ^79^, ^96^, ^97^, ^98^, ^99^, ^100^, ^101^, ^102^ For the included articles, we also perform a “*Cited by*” search to find other relevant studies.

## 5 CONCLUSION

This study provides evidence that the studied antidepressants are associated with 31% (paroxetine) to 50% (venlafaxine) increase in the risk of GIB. While we observe no differences across these antidepressants, clinicians may wish to consider initially prescribing medications with the lower risk of GIB, all other factors being equal.

## AUTHOR CONTRIBUTIONS

AGL led the systematic review, performed the literature search, data abstraction and drafted the manuscript. AGT performed the literature search, data extraction, and analysis. GF, KK, TR, and KT contributed to study design, protocol development, and provided editorial review. AJ and JM provided assistance in overall study design and conduct. DCM led the research, providing oversight and guidance throughout the study, contributed to interpretation, reviewed and edited draft versions, and provided final approval as senior author. All authors reviewed and approved the final manuscript and agreed to be accountable for all aspects of the work.

## CONFLICT OF INTEREST STATEMENT

K.K. provides consulting services to Pfizer on topics unrelated to the subject matter of this research. The rest of authors of the manuscript declare no conflicts of interest.

## Supporting information


**Data S1.** PRISMA 2020 Checklist. Risk of GIB by antidepressant

**Data S2.** Supporting Information

**Data S3.** Quality assessment of the included studies

**Data S4.** Funnel Plots for Each Antidepressant Meta‐analysis

## Data Availability

Data will be shared by the corresponding author upon request.
