# Supplementary material for: Risk of gastrointestinal bleeding by specific SSRIs and SNRIs: A systematic review and meta‐analysis
Source: Br J Clin Pharmacol. 2025 Dec 29;92(3):793–808. doi: 10.1002/bcp.70432 (PMC12930022; doi:10.1002/bcp.70432)
Supplement: Supplementary file 2 — Data S2. Supporting Information [file BCP-92-793-s001.docx]

**Table S1a**. PubMed Search Strategy for each antidepressant drug and antidepressants class.

| **Search** | **Records** |
| --- | --- |
| ((Antidepressant) AND (gastrointestinal bleed*)) OR ((Antidepressant) AND (gastrointestinal hemorrhage [MeSH Terms])) | 196 |
| ((Selective Serotonin Reuptake Inhibitor) AND (gastrointestinal bleed*)) OR ((Selective Serotonin Reuptake Inhibitor) AND (gastrointestinal hemorrhage [MeSH Terms])) | 205 |
| ((Serotonin and norepinephrine reuptake inhibitor) AND (gastrointestinal bleed*)) OR ((Selective Norepinephrine Reuptake Inhibitor) AND (gastrointestinal hemorrhage [MeSH Terms])) | 23 |
| ((Serotonin and noradrenaline reuptake inhibitor) AND (gastrointestinal bleed*)) OR ((Selective Noradrenaline Reuptake Inhibitor) AND (gastrointestinal hemorrhage [MeSH Terms])) | 23 |
| ((SSRI) AND (gastrointestinal bleed*)) | 68 |
| ((SNRI) AND (gastrointestinal bleed*)) | 19 |
| ((duloxetine) AND (gastrointestinal bleed*)) | 6 |
| ((escitalopram) AND (gastrointestinal bleed*)) | 6 |
| ((citalopram) AND (gastrointestinal bleed*)) | 11 |
| ((paroxetine) AND (gastrointestinal bleed*)) | 22 |
| ((fluoxetine) AND (gastrointestinal bleed*)) | 22 |
| ((fluvoxamine) AND (gastrointestinal bleed*)) | 7 |
| ((sertraline) AND (gastrointestinal bleed*)) | 12 |
| ((mirtazapine) AND (gastrointestinal bleed*)) | 16 |
| ((venlafaxine) AND (gastrointestinal bleed*)) | 15 |
| ((desvenlafaxine) AND (gastrointestinal bleed*)) | 1 |
| #1 OR #2 OR #3 OR #4 OR #5 OR #6 OR #7 OR #8 OR #9 OR #10 OR #11 OR #12 OR #13 OR #14 OR #15 OR #16 OR #17 OR #18 OR #19 OR #20 OR #21 OR #22 OR #23 | 298 |

From inception to October 31^st^ 2025. No restrictions.

**Table S1b**. EMBASE Search Strategy for each antidepressant drug

| **Main term** | **Search text** | **Records retrieved** |
| --- | --- | --- |
| Duloxetine | ('duloxetine'/exp OR ' duloxetine ') AND ('gastrointestinal hemorrhage'/exp OR 'gastrointestinal hemorrhage') | 199 |
| Escitalopram | ('escitalopram'/exp OR 'escitalopram') AND ('gastrointestinal hemorrhage'/exp OR 'gastrointestinal hemorrhage') | 201 |
| Citalopram | ('citalopram'/exp OR 'citalopram') AND ('gastrointestinal hemorrhage'/exp OR 'gastrointestinal hemorrhage') | 290 |
| Paroxetine | ('paroxetine'/exp OR 'paroxetine') AND ('gastrointestinal hemorrhage'/exp OR 'gastrointestinal hemorrhage') | 324 |
| Fluoxetine | ('fluoxetine'/exp OR 'fluoxetine') AND ('gastrointestinal hemorrhage'/exp OR 'gastrointestinal hemorrhage') | 379 |
| Fluvoxamine | ('fluvoxamine'/exp OR 'fluvoxamine') AND ('gastrointestinal hemorrhage'/exp OR 'gastrointestinal hemorrhage') | 154 |
| Sertraline | ('sertraline'/exp OR 'sertraline') AND ('gastrointestinal hemorrhage'/exp OR 'gastrointestinal hemorrhage') | 295 |
| Mirtazapine | ('mirtazapine'/exp OR 'mirtazapine') AND ('gastrointestinal hemorrhage'/exp OR 'gastrointestinal hemorrhage') | 203 |
| Venlafaxine | ('venlafaxine'/exp OR 'venlafaxine') AND ('gastrointestinal hemorrhage'/exp OR 'gastrointestinal hemorrhage') | 271 |
| Desvenlafaxine | ('desvenlafaxine'/exp OR 'desvenlafaxine') AND ('gastrointestinal hemorrhage'/exp OR 'gastrointestinal hemorrhage') | 27 |
| Combined | #1 OR #2 OR #3 OR #4 OR #5 OR #6 OR #7 OR #8 OR #9 OR #10 | 920 |

From inception to June 30^th^ 2025. No restrictions.

**Table S1c**. Citations of the included studies.

| **Included articles** | **PubMed Citations*** | **Web of Science Citations** | **Full text review** |
| --- | --- | --- | --- |
| Barbui 2009 | 7 | 25 | 3 |
| Carvajal 2011 | 8 | 24 | 1 |
| Chang 2022 | 5 | 12 | 1 |
| Coupland 2018 | 59 | 74 | 4 |
| Dall 2009 | 25 | 80 | 6 |
| De Abajo 1999 | 109 | 290 | 12 |
| De Abajo 2008 | 46 | 146 | 18 |
| Forgerini 2023 | 1 | 2 | 0 |
| Kurdyak 2005 | 21 | 41 | 5 |
| Li 2024 | 0 | 1 | 0 |
| Magavern 2023 | 1 | 5 | 0 |
| Opatrny 2008 | 26 | 76 | 2 |
| Schelleman 2011 | 34 | 69 | 7 |
| Verdel 2011 | 15 | 49 | 1 |
| Vidal 2008 | 21 | 52 | 4 |
| Wang 2014 | 23 | 73 | 4 |
| Wessinger 2006 | 28 | 78 | 7 |
| AFFINITY 2020 | 46 | 96 | 1 |
| EFFECTS 2020 | 54 | 109 | 6 |
| FOCUS 2019 | 102 | 209 | 4 |

* A total of 33 articles were reviewed full text. There were four systematic review and meta-analysis and all included studies (n= 14) were checked. No articles were included from the Cited by strategy neither from PubMed nor Web of Science.
