# Supplementary material for: Risk of gastrointestinal bleeding by specific SSRIs and SNRIs: A systematic review and meta‐analysis
Source: Br J Clin Pharmacol. 2025 Dec 29;92(3):793–808. doi: 10.1002/bcp.70432 (PMC12930022; doi:10.1002/bcp.70432)
Supplement: Supplementary file 3 — Data S3. Quality assessment of the included studies [file BCP-92-793-s002.docx]

**Table S3.** Quality assessment of the included studies

S3 A. Case control studies Newcastle - Ottawa quality assessment

| Case control studies | Selection | | | | Comparability | Exposure | | | TOTAL |
| --- | --- | --- | --- | --- | --- | --- | --- | --- | --- |
|  | Case definition | Representativeness | Selection of controls | Definition of controls |  | Ascertainment | Same for cases and controls | Non-response rate | * |
| Barbui 2009^1^ | * | * | * | * | ** | * | * | NA | 8 |
| Carvajal 2011^2^ | * | * | * | * | ** | * | * | NA | 8 |
| Dall 2009^3^ | * | * | * | * | ** | * | * | NA | 7 |
| De Abajo 1999^4^ | * | * | X | X | ** | * | * | NA | 6 |
| De Abajo 2008^5^ | * | * | X | X | ** | * | * | NA | 6 |
| Kurdyak 2005^6^ | * | * | X | X | ** | * | * | NA | 6 |
| Li 2024^7^ | * | * | * | X | ** | * | * | NA | 7 |
| Forgerini 2023^8^ | * | * | * | * | ** | * | * | NA | 8 |
| Opatrny 2008^9^ | * | * | * | X | ** | * | * | NA | 7 |
| Schelleman 2011^10^ | * | * | * | * | * | * | * | NA | 7 |
| Verdel 2011^11^ | * | * | * | * | ** | * | * | NA | 8 |
| Vidal 2008^12^ | * | * | * | * | ** | * | * | NA | 8 |
| Wang 2014^13^ | * | * | * | * | ** | * | * | NA | 8 |
| Wessinger 2006^14^ | * | * | * | * | * | * | * | NA | 7 |

NA not available;

**S3B**. Cohort studies Newcastle - Ottawa quality assessment

| Case control studies | Selection | | | | Comparability | Outcome | | | TOTAL |
| --- | --- | --- | --- | --- | --- | --- | --- | --- | --- |
|  | Representative exposed | Selection non exposed | Ascertainment of exposure | Outcome not present at start |  | Assessment of outcome | Follow-up length | Adequacy of follow-up |  |
| Chang 2022^15^ | * |  | * |  | * | * | * | * | 6 |
| Coupland 2018^16^ | * | * | * | * | ** | * | * | * | 9 |
| Magavern 2023^17^ | * | * | * |  | ** | * | * |  | 7 |

**S3C**. Randomized Clinical trials – Cochrane Risk of Bias 2 (RoB 2) tool

| **RCT** | **Experimental** | **Comparator** | **Outcome** | **Randomization** | **Deviation from intervention** | **Missing data** | **Measuring the outcome** | **Selection of the reported results** | **Overall bias** |
| --- | --- | --- | --- | --- | --- | --- | --- | --- | --- |
| AFFINITY^18^ | Fluoxetine | Placebo | Functional independence (mRS) | Low | Low | Low | Low | Low | Low |
| EFFECTS^19^ | Fluoxetine | Placebo | Functional independence (mRS) | Low | Low | Low | Low | Low | Low |
| FOCUS^20^ | Fluoxetine | Placebo | Functional independence (mRS) | Low | Low | Low | Some concerns | Low | Some concerns |

RCT: randomized controlled trial; mRS: modified Rankin Scale

## References

1. Barbui C, Andretta M, De Vitis G, et al. Antidepressant Drug Prescription and Risk of Abnormal Bleeding: A Case-Control Study. *J Clin Psychopharmacol*. 2009;29(1):33-38. doi:10.1097/JCP.0b013e3181929f7a

2. Carvajal A, Ortega S, Del Olmo L, et al. Selective Serotonin Reuptake Inhibitors and Gastrointestinal Bleeding: A Case-Control Study. Wright JM, ed. *PLoS ONE*. 2011;6(5):e19819. doi:10.1371/journal.pone.0019819

3. Dall M, Schaffalitzky De Muckadell OB, Lassen AT, Hansen JM, Hallas J. An Association Between Selective Serotonin Reuptake Inhibitor Use and Serious Upper Gastrointestinal Bleeding. *Clin Gastroenterol Hepatol*. 2009;7(12):1314-1321. doi:10.1016/j.cgh.2009.08.019

4. De Abajo FJ, Rodriguez LAG, Montero D. Association between selective serotonin reuptake inhibitors and upper gastrointestinal bleeding: population based case-control study. *BMJ*. 1999;319(7217):1106-1109. doi:10.1136/bmj.319.7217.1106

5. De Abajo FJ, García-Rodríguez LA. Risk of Upper Gastrointestinal Tract Bleeding Associated With Selective Serotonin Reuptake Inhibitors and Venlafaxine Therapy: Interaction With Nonsteroidal Anti-inflammatory Drugs and Effect of Acid-Suppressing Agents. *Arch Gen Psychiatry*. 2008;65(7):795. doi:10.1001/archpsyc.65.7.795

6. Kurdyak PA, Juurlink DN, Kopp A, Herrmann N, Mamdani MM. Antidepressants, Warfarin, and the Risk of Hemorrhage: *J Clin Psychopharmacol*. 2005;25(6):561-564. doi:10.1097/01.jcp.0000186869.67418.bc

7. Li YH, Hang LW, Muo CH, Chen SJ, Chen PC. Dose of selective serotonin reuptake inhibitors and risk of upper gastrointestinal bleeding in older adults. *J Psychopharmacol (Oxf)*. 2024;38(2):137-144. doi:10.1177/02698811231218955

8. Forgerini M, Schiavo G, Urbano G, et al. Use of Antidepressants and the Risk of Upper Gastrointestinal Tract Bleeding: A Case-control Study. *Clin Ther*. 2023;45(7):e159-e166. doi:10.1016/j.clinthera.2023.04.010

9. Opatrny L, Delaney JA ‘Chris’, Suissa S. Gastro‐intestinal haemorrhage risks of selective serotonin receptor antagonist therapy: a new look. *Br J Clin Pharmacol*. 2008;66(1):76-81. doi:10.1111/j.1365-2125.2008.03154.x

10. Schelleman H, Brensinger CM, Bilker WB, Hennessy S. Antidepressant-Warfarin Interaction and Associated Gastrointestinal Bleeding Risk in a Case-Control Study. Laks J, ed. *PLoS ONE*. 2011;6(6):e21447. doi:10.1371/journal.pone.0021447

11. Verdel BM, Souverein PC, Meenks SD, Heerdink ER, Leufkens HGM, Egberts TCG. Use of Serotonergic Drugs and the Risk of Bleeding. *Clin Pharmacol Ther*. 2011;89(1):89-96. doi:10.1038/clpt.2010.240

12. Vidal X, Ibáñez L, Vendrell L, Conforti A, Laporte, JR. Risk of Upper Gastrointestinal Bleeding and the Degree of Serotonin Reuptake Inhibition by Antidepressants: A Case-Control Study. *Drug Saf*. 2008;31(2):159-168. doi:10.2165/00002018-200831020-00005

13. Wang YP, Chen YT, Tsai CF, et al. Short-Term Use of Serotonin Reuptake Inhibitors and Risk of Upper Gastrointestinal Bleeding. *Am J Psychiatry*. 2014;171(1):54-61. doi:10.1176/appi.ajp.2013.12111467

14. Wessinger S, Kaplan M, Choi L, et al. Increased use of selective serotonin reuptake inhibitors in patients admitted with gastrointestinal haemorrhage: a multicentre retrospective analysis. *Aliment Pharmacol Ther*. 2006;23(7):937-944. doi:10.1111/j.1365-2036.2006.02859.x

15. Chang KH, Chen CM, Wang CL, et al. Major Bleeding Risk in Patients With Non-valvular Atrial Fibrillation Concurrently Taking Direct Oral Anticoagulants and Antidepressants. *Front Aging Neurosci*. 2022;14:791285. doi:10.3389/fnagi.2022.791285

16. Coupland C, Hill T, Morriss R, Moore M, Arthur A, Hippisley-Cox J. Antidepressant use and risk of adverse outcomes in people aged 20–64 years: cohort study using a primary care database. *BMC Med*. 2018;16(1):36. doi:10.1186/s12916-018-1022-x

17. Magavern EF, Van Heel DA, Genes & Health Research Team, Smedley D, Caulfield MJ. CYP2C19 loss‐of‐function alleles are not associated with higher prevalence of gastrointestinal bleeds in those who have been prescribed antidepressants: Analysis in a British‐South Asian cohort. *Br J Clin Pharmacol*. 2023;89(11):3432-3438. doi:10.1111/bcp.15762

18. Hankey GJ, Hackett ML, Almeida OP, et al. Safety and efficacy of fluoxetine on functional outcome after acute stroke (AFFINITY): a randomised, double-blind, placebo-controlled trial. *Lancet Neurol*. 2020;19(8):651-660. doi:10.1016/s1474-4422(20)30207-6

19. Lundström E, Isaksson E, Näsman P, et al. Safety and efficacy of fluoxetine on functional recovery after acute stroke (EFFECTS): a randomised, double-blind, placebo-controlled trial. *Lancet Neurol*. 2020;19(8):661-669. doi:10.1016/s1474-4422(20)30219-2

20. Dennis M, Mead G, Forbes J, et al. Effects of fluoxetine on functional outcomes after acute stroke (FOCUS): a pragmatic, double-blind, randomised, controlled trial. *The Lancet*. 2019;393(10168):265-274. doi:10.1016/s0140-6736(18)32823-x
