# Supplementary material for: Risk of gastrointestinal bleeding by specific SSRIs and SNRIs: A systematic review and meta‐analysis
Source: Br J Clin Pharmacol. 2025 Dec 29;92(3):793–808. doi: 10.1002/bcp.70432 (PMC12930022; doi:10.1002/bcp.70432)
Supplement: Supplementary file 4 — Data S4. Funnel Plots for Each Antidepressant Meta‐analysis [file BCP-92-793-s004.docx]

Supplemental S4. Funnel Plots for Each Antidepressant Meta-analysis

**S4a.** Funnel Plot for Citalopram
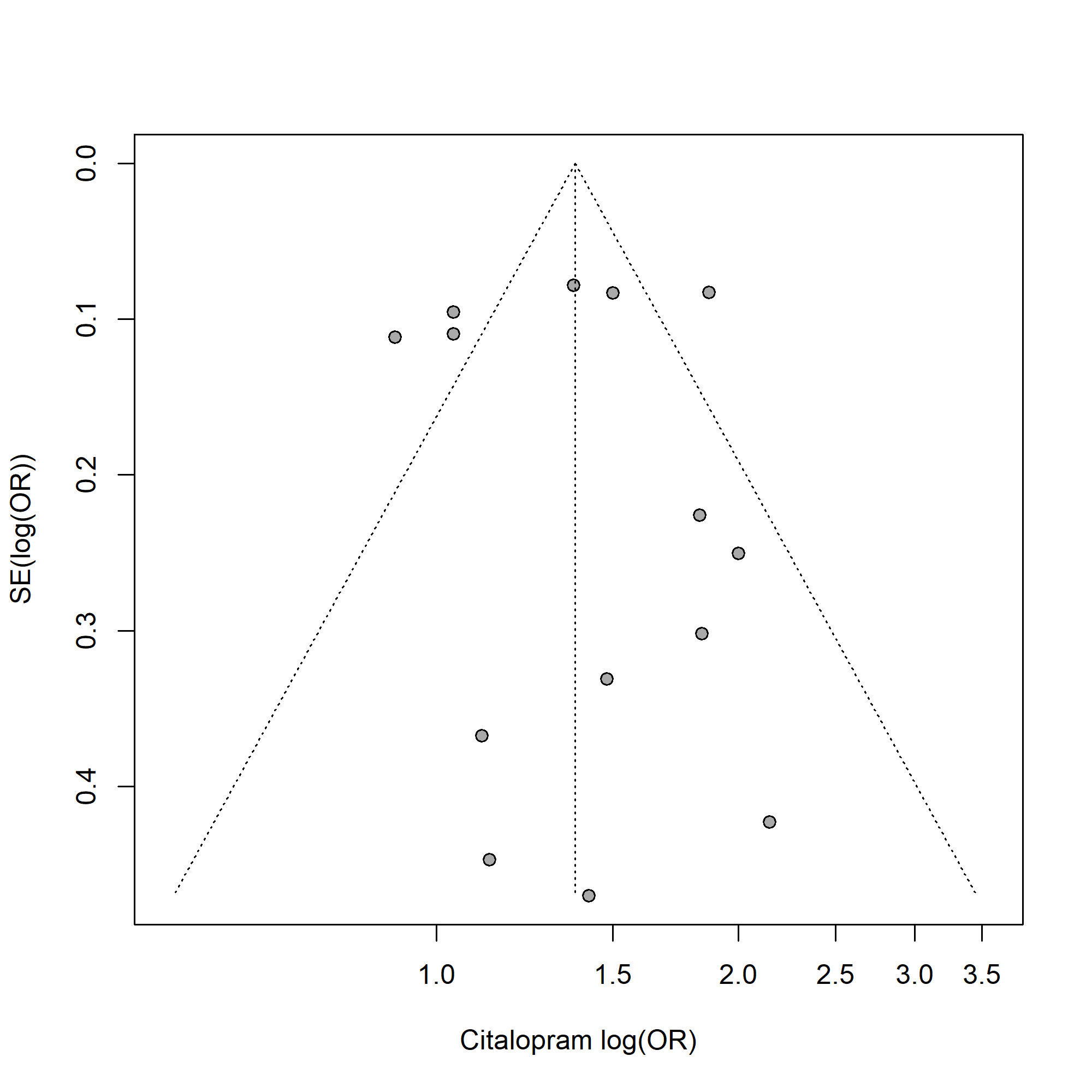


Visual inspection of the funnel plot for citalopram did not suggest marked asymmetry. Egger’s linear regression test for funnel plot asymmetry was non‑significant (t = 0.30, df = 12, p = 0.7663), with a bias estimate of 0.308 (SE = 1.013) and residual heterogeneity variance τ² = 4.125. These findings indicate no statistical evidence of small‑study effects or publication bias.

**S4b.** Funnel plot for Duloxetine**
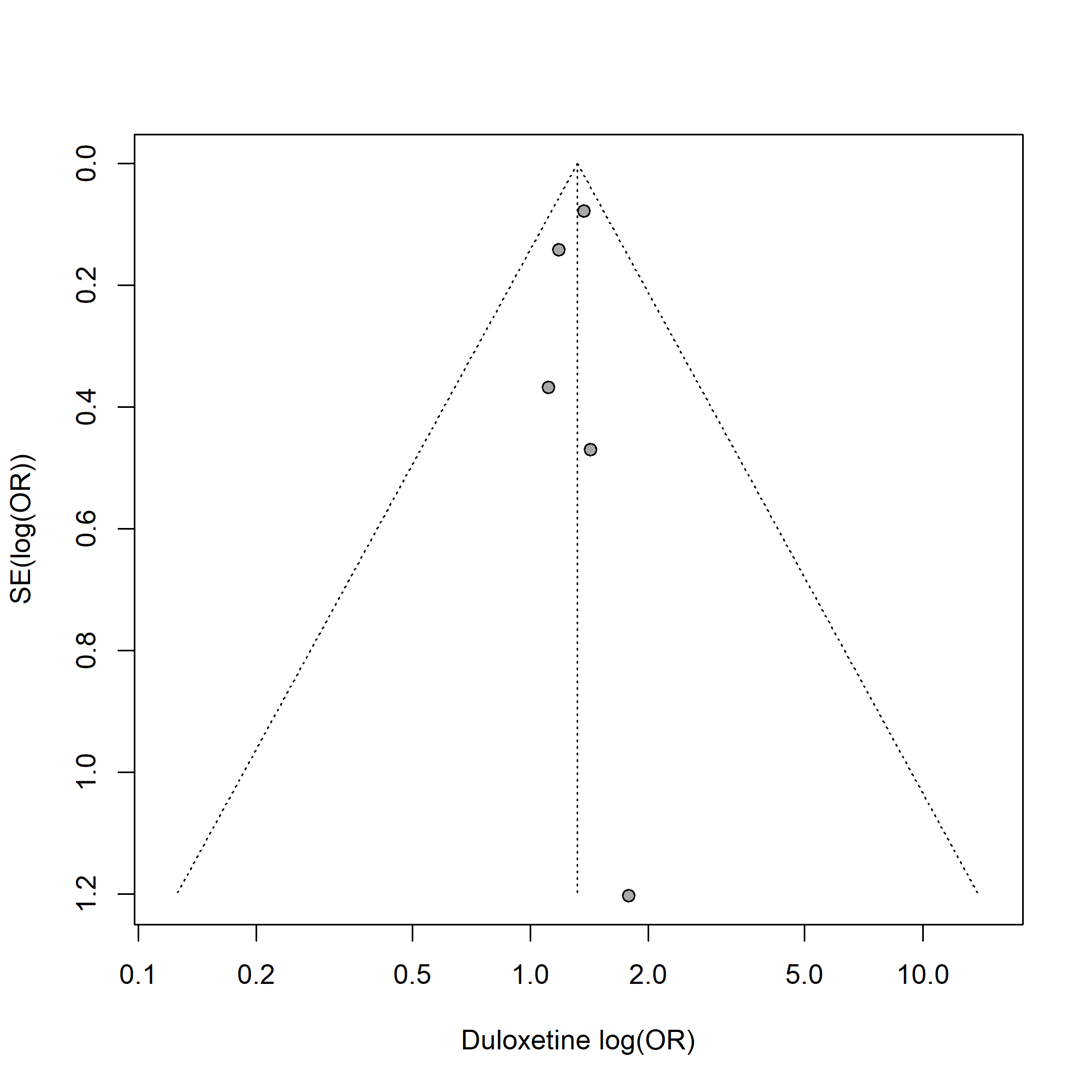
**

The funnel plot for duloxetine appeared approximately symmetric around the pooled effect estimate. Because fewer than ten studies were available (k = 5), Egger’s regression test for funnel plot asymmetry was not performed, in line with guidance that such tests are underpowered and unreliable with small numbers of studies. Although no formal statistical assessment of publication bias for duloxetine, visual inspection does not suggest substantial asymmetry.

**S4c.** Funnel plot for Escitalopram


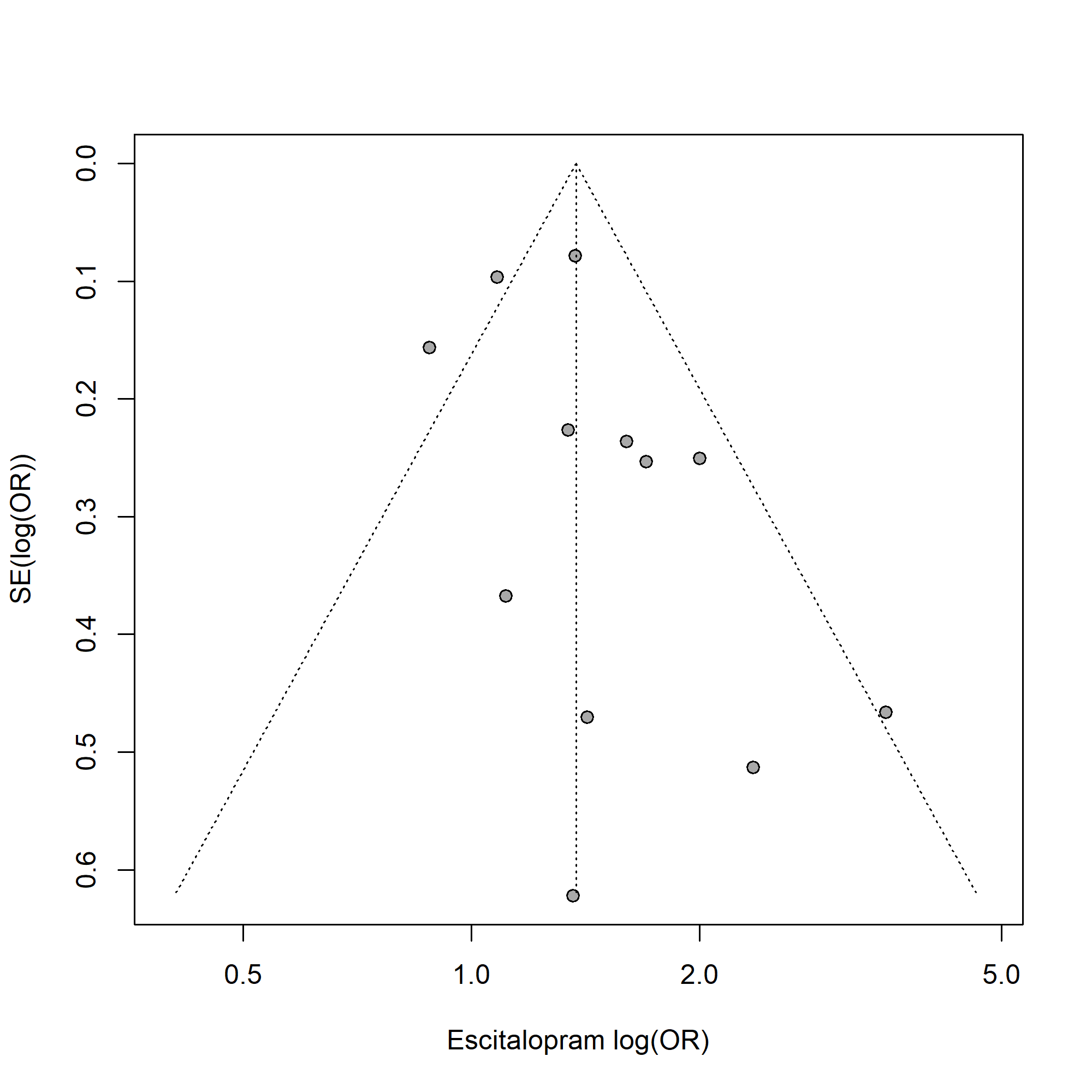


The escitalopram funnel plot showed an approximately symmetric distribution of effect sizes. Egger’s regression test was not statistically significant (t = 1.50, df = 10, p = 0.1646), with a bias estimate of 0.984 (SE = 0.656) and τ² = 1.737. Taking together, these results provide no evidence of important small‑study effects.

**S4d.** Funnel plot for Fluoxetine


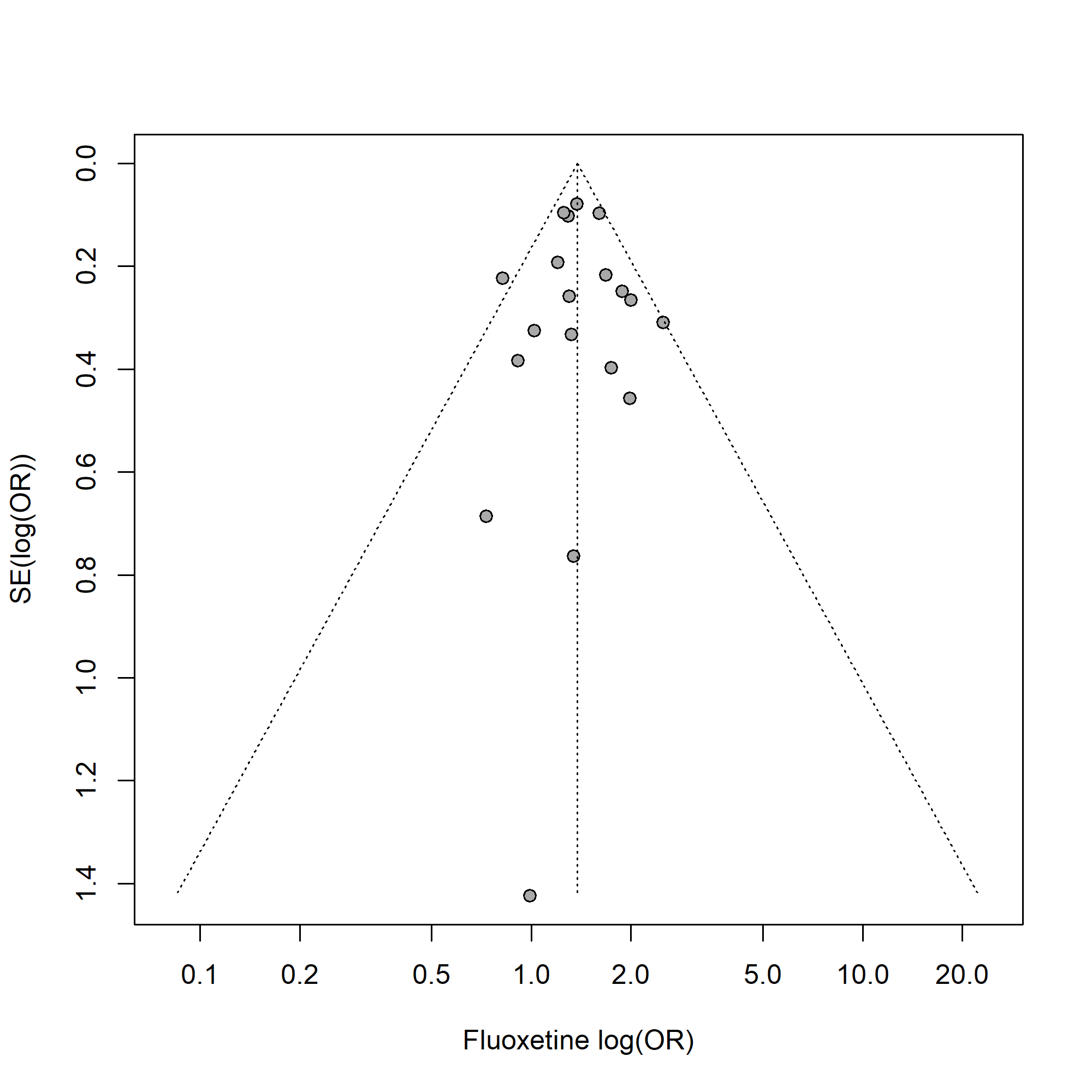


For fluoxetine, the funnel plot appeared broadly symmetric around the pooled odds ratio. Egger’s linear regression test did not indicate significant funnel plot asymmetry (t = 0.06, df = 17, p = 0.9540). The estimated bias was 0.026 (SE = 0.445), with τ² = 1.286. These findings suggest no evidence of publication bias among studies of fluoxetine and GIB risk.

**S4e.** Funnel plot for Fluvoxamine


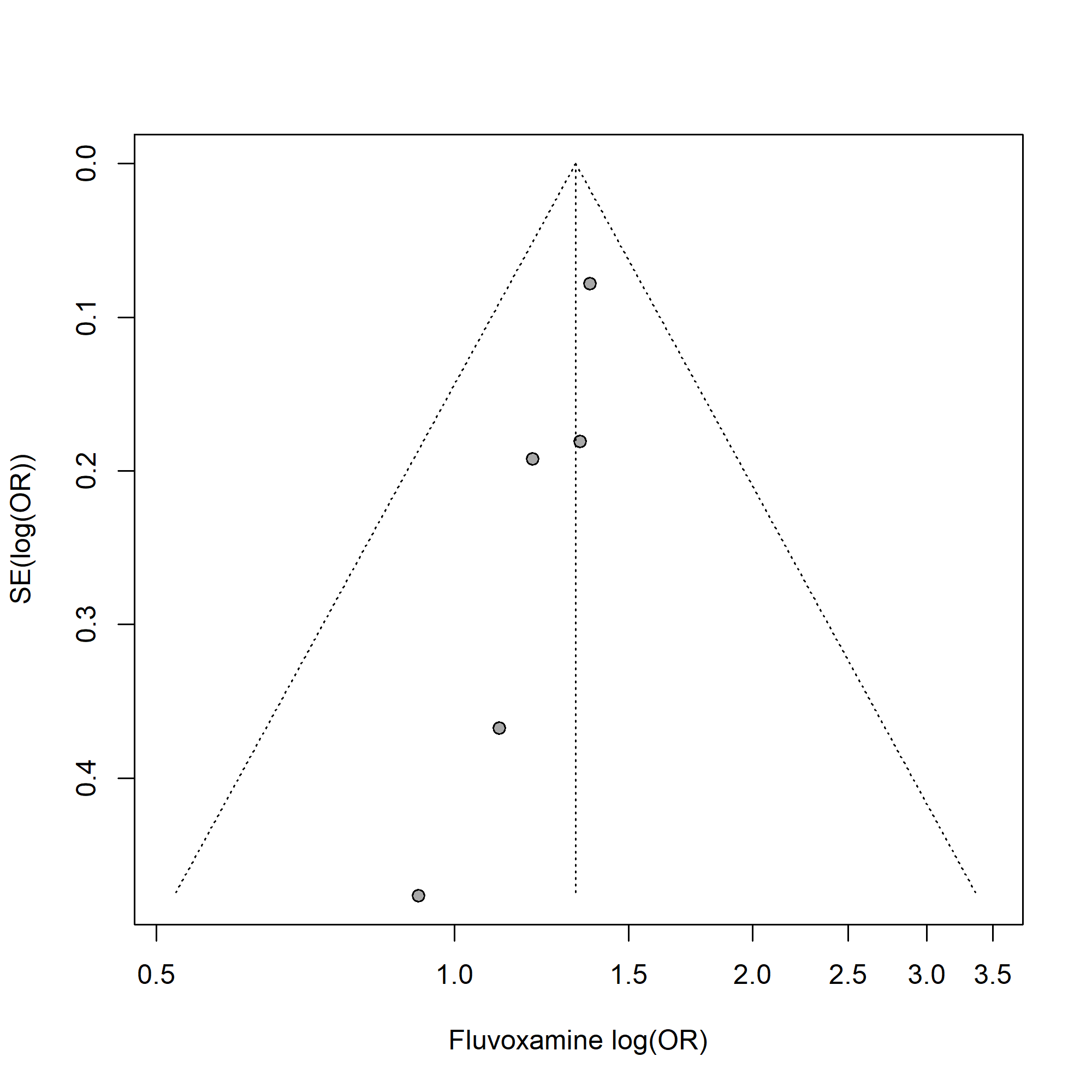


The funnel plot for fluvoxamine did not show obvious asymmetry on visual inspection. However, because only five studies were available, formal statistical tests for small‑study effects (such as Egger’s regression) were not conducted, as recommended when k < 10. Accordingly, conclusions about publication bias rely primarily on visual assessment of the funnel plot.

**S4f.** Funnel plot for Paroxetine


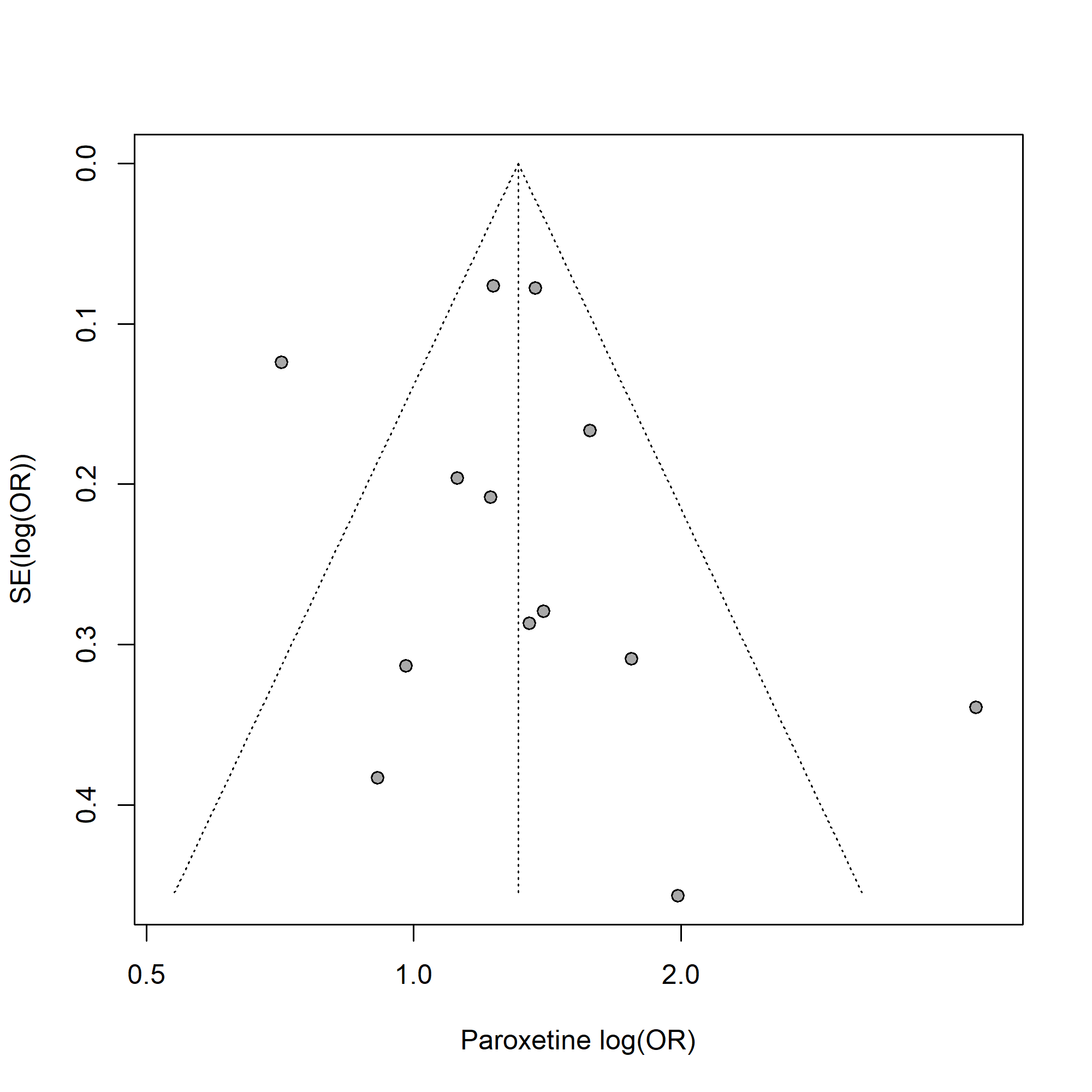


The funnel plot for paroxetine appeared reasonably symmetric. Egger’s test for funnel plot asymmetry was non‑significant (t = 0.70, df = 11, p = 0.4965), with a bias estimate of 0.683 (SE = 0.971) and τ² = 3.619. These results do not support the presence of substantial small‑study effects.

**S4g.** Funnel plot for Sertraline


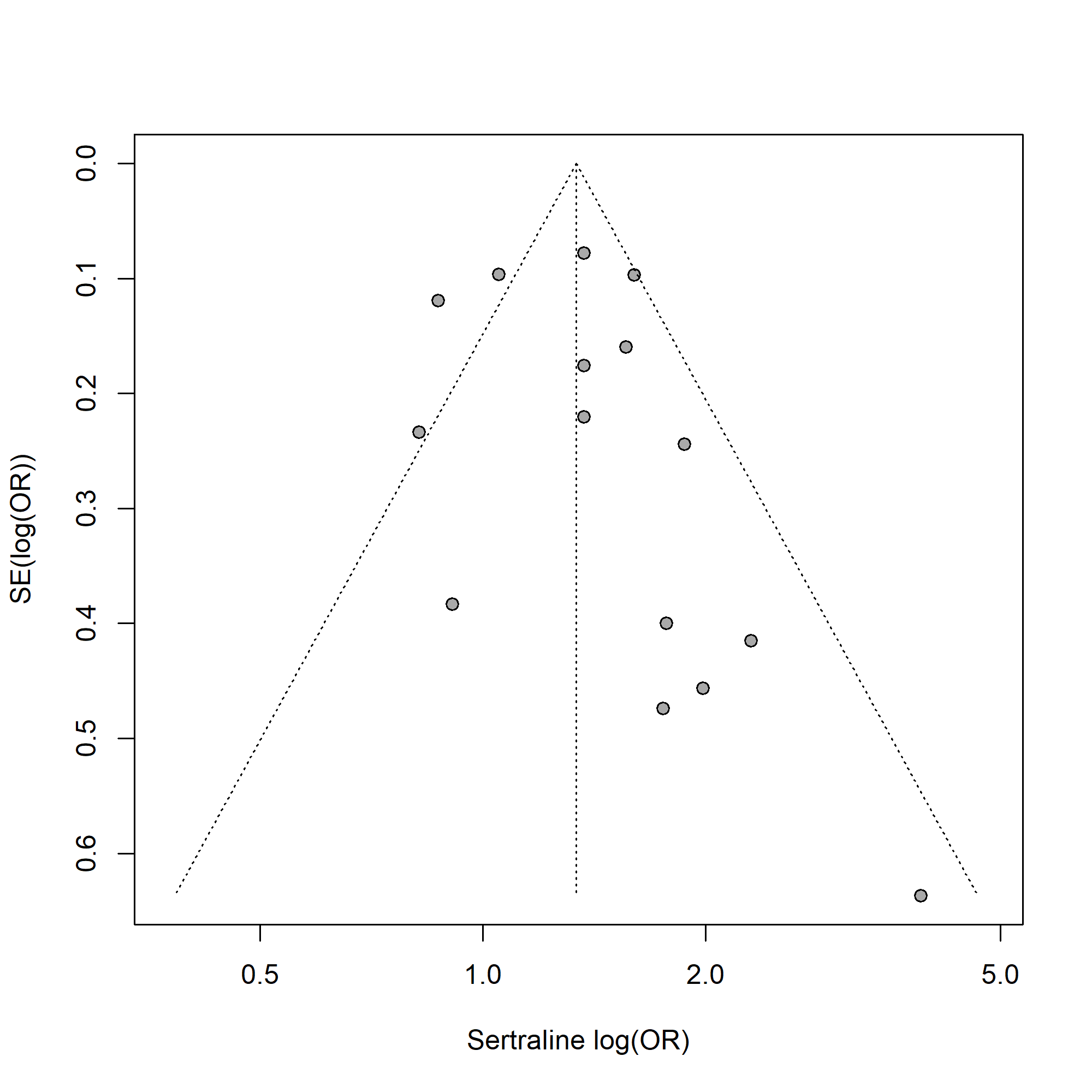


Inspection of the funnel plot for sertraline suggested approximate symmetry around the pooled effect. This impression was supported by Egger’s regression test, which was not statistically significant (t = 1.02, df = 13, p = 0.3277). The estimated bias was 0.780 (SE = 0.767), with τ² = 2.598. Overall, there was no indication of meaningful publication bias in the sertraline analyses.

**S4h.** Funnel plot for Venlafaxine


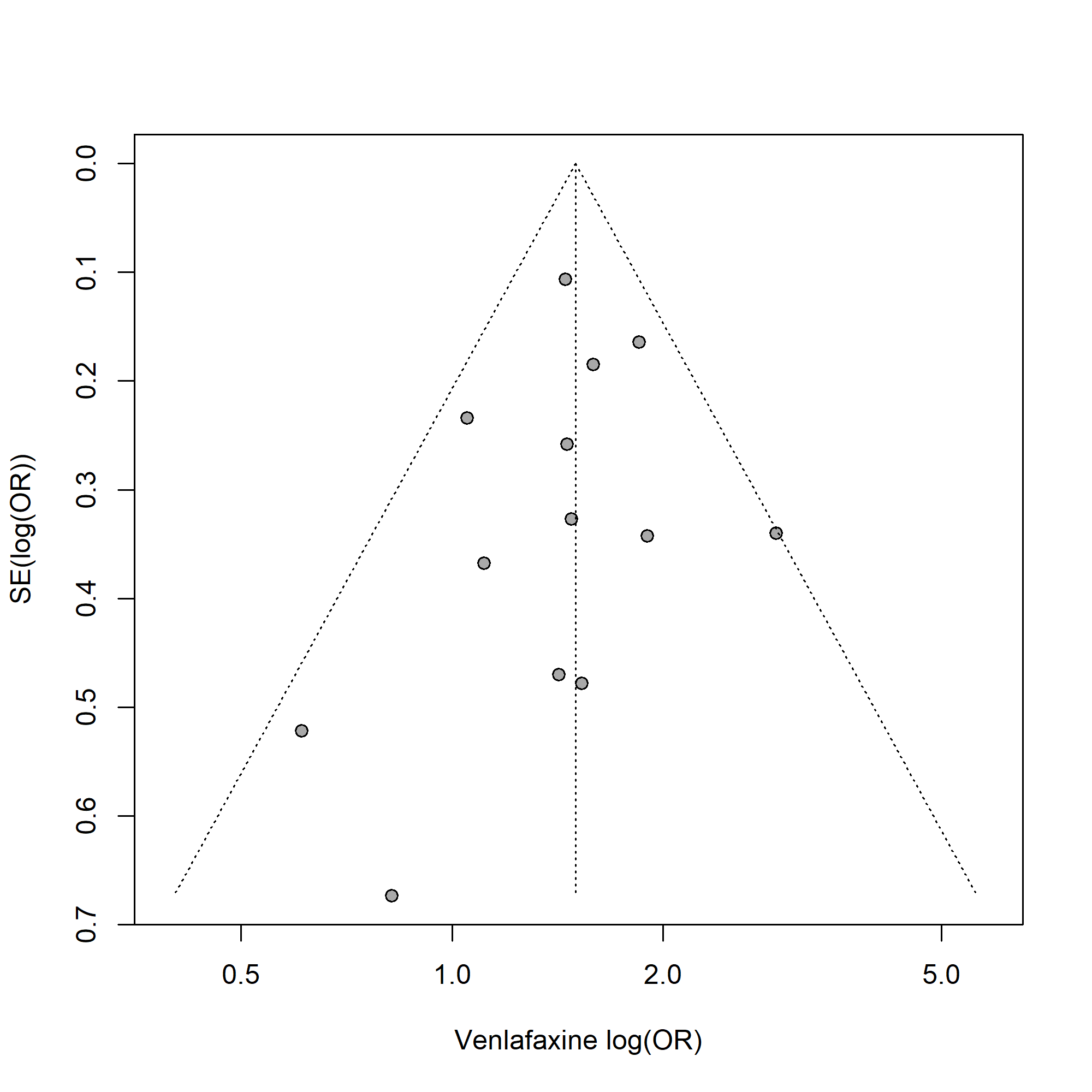


The venlafaxine funnel plot also appeared symmetric. Egger’s test did not reveal significant funnel plot asymmetry (t = –0.70, df = 11, p = 0.4999), with a bias estimate of –0.417 (SE = 0.597) and τ² = 1.121. Both the visual and statistical assessments argue against important small‑study effects among studies of venlafaxine.
